# Identification of Retinal Biomarkers in Alzheimer’s Disease Using Optical Coherence Tomography: Recent Insights, Challenges, and Opportunities

**DOI:** 10.3390/jcm8070996

**Published:** 2019-07-09

**Authors:** Delia Cabrera DeBuc, Magdalena Gaca-Wysocka, Andrzej Grzybowski, Piotr Kanclerz

**Affiliations:** 1 Bascom Palmer Eye Institute, Department of Ophthalmology, Miller School of Medicine, University of Miami, Miami, FL 33136, USA; 2 Poznań City Hospital, 61-285 Poznań, Poland; 3 Department of Ophthalmology, University of Warmia and Mazury, 10-082 Olsztyn, Poland; 4 Institute for Research in Ophthalmology, Foundation for Ophthalmology Development, 60-554 Poznan, Poland; 5 Private Practice, 80-822 Gdańsk, Poland

**Keywords:** Alzheimer’s disease, central nervous system diseases, ganglion cell layer, optical coherence tomography, retinal nerve fiber layer

## Abstract

This review will highlight recent insights into measuring retinal structure in Alzheimer’s disease (AD). A growing body of evidence indicates that disturbances in retinal blood flow and structure are related to cognitive function, which can severely impair vision. Optical coherence tomography (OCT) is an optical imaging technology that may allow researchers and physicians to gain deeper insights into retinal morphology and clarify the impact of AD on retinal health and function. Direct and noninvasive measurement of retinal morphology using OCT has provided useful diagnostic and therapeutic indications in several central nervous system (CNS) diseases, including AD, multiple sclerosis, and Parkinson disease. Despite several limitations, morphology assessment in the retinal layers is a significant advancement in the understanding of ocular diseases. Nevertheless, additional studies are required to validate the use of OCT in AD and its complications in the eye.

## 1. Introduction

Optical coherence tomography (OCT) has transformed the diagnosis and treatment of ocular disease. Currently, OCT is one of the most widely used ophthalmic decision-making technologies [1]. OCT uses retroreflected light to provide micron-resolution, cross-sectional images of biological tissues. The first experimental OCT device developed to image the human retina in vivo was introduced in 1991 [2]. OCT has become a compelling medical imaging technology, particularly in ophthalmology, as it allows achieving the cross-sectional structure of the retina and anterior eye with higher resolutions than any other non-invasive imaging modality [2]. Even commercially available OCT systems exhibit extremely high resolution, typically in the micrometer-scale resolution; and the resulting images can be analyzed both qualitatively and objectively. The recent introduction of OCT angiography (OCTA) and wide-field imaging in clinical practice has led to significant advancements in retinal disease understanding and treatment [1]. Further developments of OCT technology may impact eye disease diagnosis and improve the management of the major clinical and public health problems associated with visual impairment. Also, there is growing evidence to incorporate the OCT technology into clinical settings managing cerebrovascular and neural diseases [3].

Alzheimer’s disease (AD) is the most prevalent chronic neurodegenerative disorder and the cause of dementia in the elderly [4]. The global prevalence of AD is 36 million people and is estimated to double every 20 years, reaching 115 million in 2050 [5]. The pathologic findings typical for AD are beta-amyloid (Aβ) plaques, neurofibrillary tangles (NFTs), and reactive gliosis [6]. Recent studies have shown that AD initiates decades before it is clinically expressed [7,8,9,10,11,12]. Therefore, it could be possible to identify individuals who will develop AD before the early symptoms appear, and potentially to employ prevention in high-risk patients [4,5,7,8,9,10,11,12]. In clinical practice, the diagnosis of AD is based on cognitive evaluation; such an approach might be insufficient in individuals with much brain or cognitive reserve. Also, evaluation of brain biochemistry and anatomy using molecular markers or neuroimaging modalities are not surrogates for cognitive processing, nor psychological function. Currently used diagnostic techniques, which include neuroimaging (e.g., magnetic resonance imaging (MRI) and positron emission tomography (PET)) or cerebrospinal fluid protein levels (e.g., tau and Aβ), are costly or relatively invasive. Also, these techniques present low specificity and are not readily accessible to the majority of clinicians and patients.

As the eye and brain share critical structural and pathogenic pathways, a non-invasive multivariate biomarker methodology using the eye may provide new insights into the onset and progression of AD. The link between eye pathology and AD has been established. In patients with AD, the visual function is commonly affected; symptoms include loss of best-corrected visual acuity, a reduction in contrast sensitivity, ocular motility abnormalities, and color vision defects [13]. It is known that the most likely locations for AD onset are parahippocampal regions, the entorhinal cortex, and hippocampus [14]. Interestingly, McKee et al. found dense AD pathology in the visual association cortex Brodmann area 19 in some cognitively intact individuals with preclinical AD, with the absence of significant pathology in the hippocampus or entorhinal cortex [15]. It was hypothesized that area 19 might confer enhanced vulnerability to neurodegeneration [15].

Recently, advances in neuro-electrophysiological tests and optical imaging have made it possible to detect specific manifestations of neurodegenerative diseases in the eye; in particular, retinal microvascular alterations with abnormal bioelectrical activity of retinal ganglion cells, photoreceptors, and the optic nerve have been associated with cognitive decline and brain alterations in relation to aging and brain abnormalities in early AD [16,17]. Also, evidence of ganglion cell loss and photoreceptor damage observed in AD patients has been reported using OCT [16,17]. Not only retinal but also choroidal thickness was found to be reduced in enhanced depth SD-OCT studies [18,19]. Based on the evidence mentioned above, researchers have even suggested that if an association can be made between the amyloid in the brain and particular manifestations in the eye, then it would be feasible to diagnose AD by a specific eye examination.

This review will highlight recent insights into measuring retinal structure in patients with AD and the identification of retinal biomarkers using commercially available OCT devices. OCT may offer an opportunity to improve the understanding of the neurobiological changes in neurodegenerative diseases such as AD and may aid to develop both diagnostic and prognostic biomarkers that can predict clinical progress. A mounting body of evidence suggests that disturbances in retinal blood flow and structure are related to cognitive function, which can severely impair vision. The OCT technology may allow researchers and physicians to gain deeper insights into retinal morphology and clarify the impact of AD on retinal health and function. This review will also focus on the challenges and opportunities associated with the applications of OCT technology to identify AD’s biomarkers in the eye.

## 2. Material and Methods

This study was exempted from approval by the Institutional Review Board from both the University of Miami and University of Warmia and Mazury, as it did not include active human subject research. Only manuscripts investigating AD’s biomarkers using commercially available OCT technology published in peer-reviewed publications between 2001 and December 2018 were considered for this review.

The online citation index service PubMed was searched using the keywords optical coherence tomography and Alzheimer’s disease. Of the articles retrieved, all publications in English and abstracts from non-English publications were reviewed. The reference lists of the analyzed articles were also considered as a potential source of information. Studies analyzing the results of OCT angiography were excluded. Additionally, considering that past studies could be of varying quality and follow different protocols to collect data, retrospective meta-analyses were not included in this review. In original research articles, the revisions considered patient selection criteria, demographics, group sizes, the characteristics of the control group recruited, the type of commercial OCT device used in the study, as well as the brain and eye screening method employed to collect the data. The data extracted are presented in a table format consisting of author(s)’ name, journal source and year of publication, patient selection criteria and sample size, and a summary of the clinical findings.

## 3. Results and Discussion

**Figure 1:**
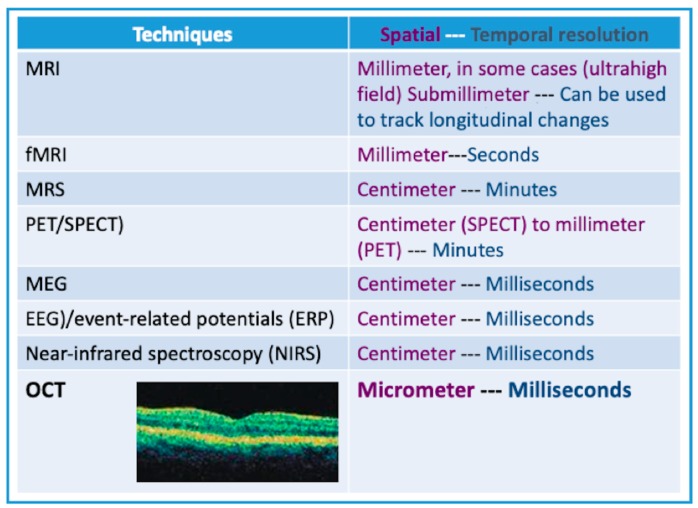
OCT’s spatial-temporal resolution vs. those of conventional clinical technologies. Magnetic resonance imaging (MRI), functional magnetic resonance imaging (fMRI), magnetic resonance spectroscopy (MRS), positron emission tomography (PET), single photon emission computed tomography (SPECT), magneto-electroencephalography (MEG), electroencephalography (EEG), optical coherence tomography (OCT).

**Figure 2:**
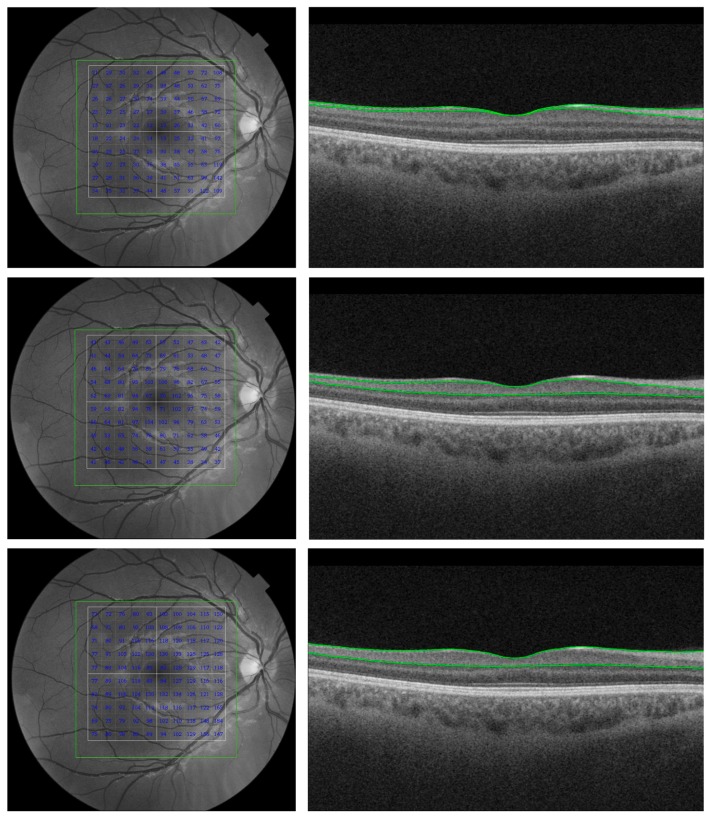
Capabilities of ocular imaging showing the retinal layer thickness measurements for the retinal nerve fiber layer (RNFL, top row), ganglion cell layer and inner plexiform layer complex (GCL + IPL, middle row), and the ganglion cell complex (GCC consisting of the RNFL + GCL + IPL, bottom row) in each 10 × 10 grid cell within a macular area of 6 × 6 mm. Images were obtained with a 3D OCT-2000 unit (software version 8.11, Topcon Corp., Tokyo, Japan). The horizontal OCT B scans (right column) reveal the corresponding boundaries (green lines) of the inner retinal layers. The scanned macular area (7 × 7 mm) is shown on the left column. Image was taken with permission from Cunha et al. [20].

**Figure 3:**
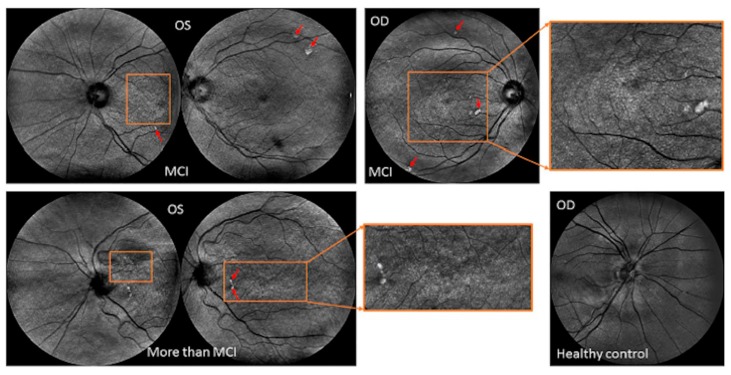
Capabilities of ocular imaging revealing drusen-like regions in the peripheral retina along with pigment dispersion noted in subjects with mild and more severe cognitive impairment. The red arrows indicate the location of the drusen and white spots observed at extramacular locations. The areas enclosed by the orange rectangles indicate the locations where pigment dispersion was observed. Abbreviations: mild cognitive impairment (MCI), oculus sinister (OS), oculus dextrus (OD).

OCT is a powerful tool, having a spatial resolution far higher than in conventional clinical technologies such as computerized tomography, ultrasound, or magnetic resonance imaging (Figure 1). This review revealed the capabilities of OCT technology to see the brain through the eye. Generally, it is possible to evaluate alterations of the optic nerve only in histological examinations. However, the transparent medium of the eye provides a unique opportunity for objective quantitative measurements and in vivo real-time images of ocular structures (Figure 2 and Figure 3).

### 3.1. General Findings

The initial search for this review found 143 studies from the PubMed database. After content analysis, 96 articles dating from 2001 to 2018 were assessed as significant. The exclusion of non-English language publications might constitute a limitation of this study; however, it is commonly applied in review articles. The articles included in this review focused on a broad range of specific objectives with retinal biomarkers being one of several. Table 1 shows the study details for the selected studies. In particular, this review revealed that limited research had focused exclusively on screening the eyes of study subjects with and with no cognitive decline using optical coherence tomography, neuropsychological tests, and in vivo neuroimaging techniques. All studies screened the eyes with the OCT technology after pupil dilation. Also, axial length and refraction data were not collected in many of the studies. Therefore, based on the potential increase of retinal thinning with greater axial length, it is unclear how this effect impacted much of results reported [21]. Fourteen studies used the time-domain Stratus OCT device (Carl Zeiss Meditec, Dublin, CA, U.S.A.). However, most of the research was conducted on spectral-domain OCT platforms including Spectralis SD-OCT (Heidelberg Engineering GmbH, Heidelberg, Germany), Cirrus HD-OCT (Carl Zeiss Meditec, Dublin, CA, U.S.A.), Topcon 3D OCT (Maestro, 1000 and 2000 Series, Topcon Medical Systems Inc., Tokyo, Japan) and RTVue-100 (Optovue Inc., Fremont, CA, USA). In general, the OCT measurements of the ganglion cell layer (GCL)-inner plexiform layer (IPL) and retinal nerve fiber layer (RNFL) present good intra-visit repeatability and inter-visit reproducibility [22]. Less than 9% of the studies used MRI or PET for the diagnosis in the AD group. In most of the studies, women were most commonly predominant in all cohorts.

The studies varied in their sample sizes and the number of participants, which ranged from 8 to 2124. In summary, the OCT results of 7148 participants were evaluated. Most of the criteria for pathological participant selection included mild cognitive impairment (MCI), subjective memory complaint, and early AD. Certain health conditions such as hypertension, diabetes, chronic heart failure, cardiac insufficiency, stroke, heart arrhythmia, age-related macular degeneration, and glaucoma were considered in the exclusion criteria in most articles. In addition to these conditions, much of studies conducted the Mini-Mental State Examination (MMSE) and other neuropsychological tests used across the National Institute on Aging (NIA) to determine the mental condition of the patients [23,24]. Three studies used the Montreal Cognitive Assessment (MoCA), along with the NIA tests and MMSE. Overall, the cognitive methods used in each study varied, which may also elucidate some of the differences. Of note, even the recent studies commonly did not follow the Advised Protocol for OCT Study Terminology and Elements (APOSTEL) recommendations [25]. These guidelines, conceived by a group of researchers and members of the International Multiple Sclerosis Visual (IMSVISUAL) consortium, were developed to highlight the essential information that should be provided when reporting quantitative OCT.

In general, the variability in the overall study design, the use of different generations of OCTs from various manufacturers with potential differences in resultant measurements, as well as the lack of standardization when analyzing OCT data within studies, makes the straightforward comparability of measurements challenging. Also, the retinal abnormalities in AD are complicated by the fact that both neurodegenerative and chronic diseases and AD are strongly age-related, and that, consequently, the indicators overlap, and attribution of retinal changes to one pathology rather than the other may be challenging. Therefore, a common trend in the studies reviewed was the analysis of retinal features between individuals along with the existing confounding variables (e.g., diabetes, hypertension, aging) that may also result in retinal changes. Moreover, much of the studies assessed in this review comprise only cross-sectional data, which is a critical limitation for the investigation of imaging biomarkers, particularly for disease progression and treatment success. Longitudinal studies will better facilitate the analyses of progressive changes, but a larger sample size might be needed to delineate disease stage differences as well as assess the sensitivity and specificity to change. Longitudinal data on retinal changes with healthy aging is also fundamental to characterize retinal abnormalities associated with cognitive impairment within a single individual over time. Ultimately, investigating associations between retinal changes and aging-related brain processes in neurologically healthy older adults could reveal whether AD-related pathologic changes occurring in the retina and central visual pathways are specific to axonal and ganglionic cell body injury.

**Figure 4:**
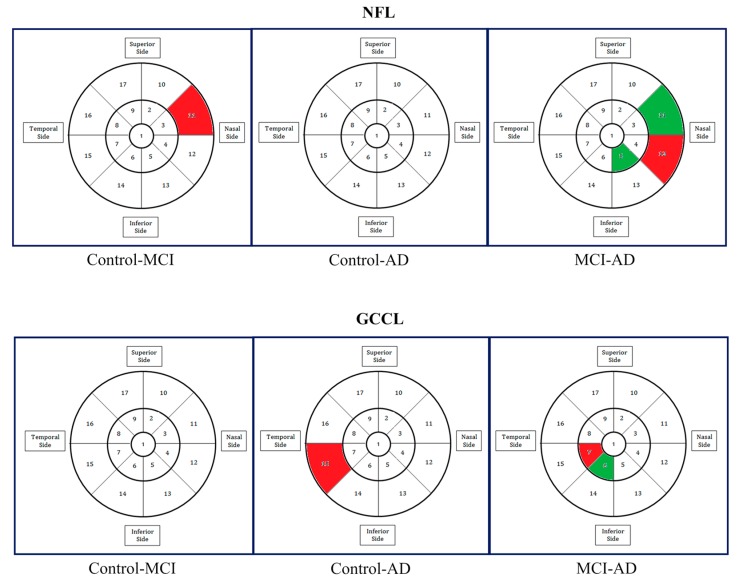
Multi-variate regression analysis results obtained after investigating the association between the NFL and GCIPL layer thicknesses (i.e., GCL + IPL complex) to the disease categories of the participants: control, mild cognitive impairment (MCI) or Alzheimer’s disease (AD) by using a quasi-least squares technique, adjusted for multiple comparisons. In this analysis, a total of 17 regional thickness measurements for both NFL and GCIPL were used. Also, note that areas in the macula were statistically significantly thinner (red) or thicker (green). (Image was taken with permission from Lad et al. [26]).

Although most reviewed studies identified retinal abnormalities in AD patients such as reduced RNFL thickness, and degeneration of retinal ganglion cells (RGC), these deficits are not AD-specific and are seen in patients with other degenerative diseases such as diabetic retinopathy, glaucoma, and multiple sclerosis. In particular, a common finding in all studies was the thinning of the peripapillary RNFL as well as macular thinning of the GCL, IPL, and the ganglion cell complex (RNFL + GCL + IPL). Interestingly, Lad et al. conducted a more complex statistical analysis and revealed areas of thinning adjacent to areas of thickening in the macula of MCI and AD patients (Figure 4), suggesting that these retinal layers might be undergoing dynamic changes during the development of AD progression [26]. Also, it was found that not only the retinal structure of patients with AD had been investigated, but that the choroid thickness measured with enhanced depth SD-OCT was also found to be reduced [18,19]. Moreover, some recent studies have reported significant choroidal thinning in patients with AD when compared with elderly subjects [27,28]. This finding may aid in the diagnosis of Alzheimer’s choroidopathy not related to age. Overall, these general findings are of extreme importance to understanding how changes in the retinal neurovascular unit reflect neurovascular pathology in the brain.

### 3.2. OCT Findings and Normative Data

**Figure 5:**
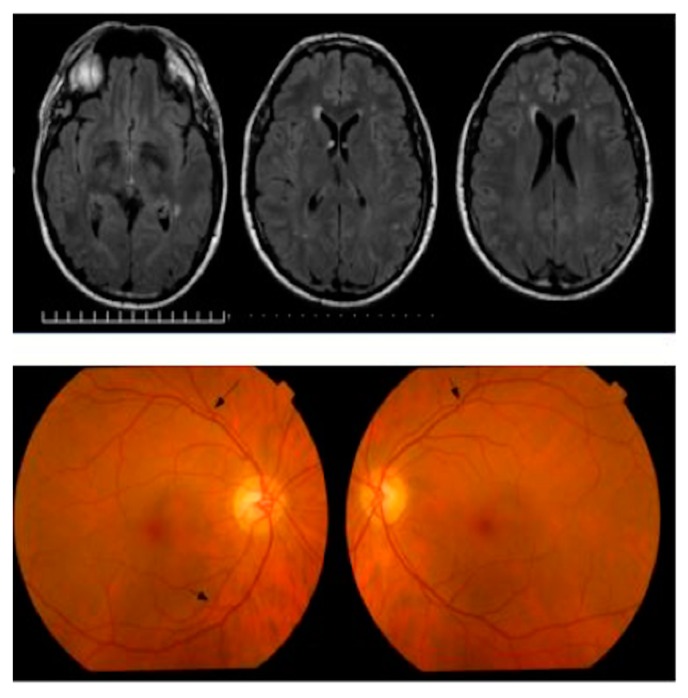
Magnetic resonance images (MRI) and fundus images from a supposedly healthy subject (male, 51 years old) with discrete focal lesions in the brain without visible or detectable ocular manifestations. TOP: MRI images showing mild to moderate white matter (WM) disease in the MRI image. The images show discrete focal lesions in anterior and posterior WM, focal confluence in posterior WM, and periventricular caps. BOTTOM: Fundus images annotated (see black arrows) showing arterio-venous crossings. The arterio-venous crossings are frequent in the average healthy population. (Image Courtesy of Valia Rodriguez at Aston University, personal communication).

Concerning normative data, it was also found that less than a third of the studies used MRI or CT screening for healthy controls. Therefore, an important consideration is to carefully collect retinal imaging data from healthy subjects without subtle brain abnormalities. As the results of elderly patients are commonly analyzed, it is unknown whether the healthy subjects included in normative databases of retinal imaging devices may have discrete focal lesions (i.e., mild signs) in the brain without visible or detectable ocular manifestations (Figure 5). Thus, caution should be exercised when considering normative data from retinal imaging, particularly if considering them for introducing new biological markers of non-ocular disease.

### 3.3. OCT Findings Concerning Neuroimaging Features and Cognitive Tests

Current efforts are focused on detecting the presymptomatic phase (MCI) of the disease during clinical evaluations of healthy brain functioning. For example, Den Haan et al. showed that retinal thickness in early-onset AD correlated with parietal cortical atrophy [30]. Disease-modifying agents might be obtainable in the future while existing therapy for Alzheimer disease comprises enhancers of cholinergic function. Although medial temporal lobe atrophy was found to be a more valuable predictor of cognition than small-vessel disease in MCI [67], it is also essential to consider that “supposedly” healthy individuals with no systemic or chronic diseases may have beta-amyloid (Aβ) plaques in their brain and still have a normal healthy cognitive function. Also, not all MCI patients convert to AD [68]. It has also been reported that identifying the transition from the asymptomatic phase to symptomatic pre-dementia phase or from the symptomatic pre-dementia phase to dementia onset in the clinical setting is a non-trivial problem [69]. This matter creates a diagnostic uncertainty for the early stage of the disease. These caveats pointed out the need to identify new clinical biomarkers that could also be valuable in individuals without traditional risk factors (e.g., age, hypertension, diabetes, smoking habits, cardiovascular disease, hyperlipidemia (cholesterol, triglycerides). Most studies did not report quadrant-specific retinal OCT abnormalities. Those that found such a correlation revealed that peripapillary RNFL thickness in AD is lower in the superior and inferior quadrants than in nasal and temporal quadrants. It is uncertain why the superior and inferior quadrants could be favorably disturbed in some studies, but it might be because retinal features such as axons and nerve fibers with bigger diameter degenerate more promptly and those features are more abundant in superior and inferior quadrants. A significant reason for the mixed results among these studies is the use of different OCT devices (e.g., time domain v. spectral domain) which appears to be the more significant variable causing heterogeneity in thickness measurements. Shi et al. reported that the inferior quadrant exhibited the strongest associations with results of cognitive tests, indicating that such results could show a higher risk to develop cognitive decline in older adults [55]. On the other hand, Uchida and associates revealed that cognitive testing scores correlated with the ellipsoid zone-retinal pigment epithelium volume [30].

Most of the studies employed the MMSE test. A drawback of the MMSE is its poor sensitivity for distinguishing MCI, which can be attributed to a lack of complexity as well as the absence of executive function items [70,71,72,73]. Its ceiling effect for healthy individuals is a common drawback that increases the likelihood that individuals in predementia phases score within the normal range (24 and above) [74,75]. MoCA is a more thought-provoking test that includes, higher-level language, executive function, and complex visuospatial processing to allow recognition of MCI with less ceiling effect [76,77]. A recent study reported that although MMSE and MoCA are more similar for dementia cases, the MoCA allocates MCI cases across a larger score range with less ceiling effect [78]. Therefore, MoCA might identify early and late MCI cases with more sensitivity than the MMSE. Also, it is crucial to consider that unusual presentation, or the existence of amnestic features in non-Alzheimer dementias could make to occasionally misdiagnosed AD as other dementias. Nonetheless, a thorough clinical history along with detailed neuroimaging assessment, and comprehensive mental evaluation will overcome this confusion.

### 3.4. OCT Findings and AD Biomarkers

The discovery of biomarkers is a difficult process that requires consideration of various factors and methods to obtain reliable biomarkers that could allow to predicting risk or response to treatment very early and with low false positive and false negative rates. To develop a biomarker-guided integrative AD modeling using precision medicine, the Alzheimer Precision Medicine Initiative group suggested applying a systematic neuropsychological and biological approach in exploratory translational neuroscience research on neurodegenerative diseases [79]. In ophthalmology, the continuous development of medical technology and methods to analyze and interpret ophthalmic data has facilitated the introduction of many retinal measures over time. For example, multiple optical-structural and functional parameters could be measured to characterize the retinal tissue, such as thickness, volume, optical scattering and polarization properties, texture measures, vasculature network metrics, blood flow dynamics, oximetry, electro-physiology metrics, fractal dimension, and lacunarity. Over the years, these retinal measures have been investigated and used as potential biomarkers of retinal abnormalities to elucidate potential applications in therapeutics [80]. Some aspects to be contemplated in the detection and assessment of biomarkers are the differences in phenotyping, the velocity of disease development in patients, the disease duration and impact of age, the subclinical ocular damage, the practicality of biomarkers to be confirmed in independent populations. Also, it should be noted that the prognosticators at the population level might not apply to all persons, and that large-scale studies are often needed. The goal would be to improve early detection by developing tools that aid in the identification of biomarkers that allow moving diagnosis backward in the temporal course.

In the search for AD biomarkers using OCT technology, more studies are necessary to assess whether choroidal, RNFL or GCL and IPL thickness represent an additional biomarker for the diagnosis and follow-up of AD pathology. The recent introduction of OCT angiography in Ophthalmology offers a novel approach to investigate retinal microvascular alterations in the foveal avascular zone and vessel density that may facilitate evaluating markers of non-perfusion and decreased capillary blood flow in the choriocapillaris as well as in the superficial and deep retinal vascular plexuses. These OCTA biomarkers could indicate microvascular network changes in the retina, mirroring changes in the cerebral microcirculation associated with cognitive function deterioration in the elderly. Feke et al. reported reduced blood flow in MCI patients in the presence of unchanged RNFL thickness [81]. Therefore, it might be presumed that blood flow abnormalities may precede the neurodegeneration in AD. Therefore, retinal hemodynamic measures could also be used as potential surrogate metrics for the cortex given the inaccessibility and the greater cost of imaging the vasculature of the cortex. Interestingly, retinal hemodynamics have not been practically explored concerning cognitive decline. Recently, contrary to previous understanding, the amyloid buildup is not the first sign of late-onset AD. Iturria-Medina et al. found that the first physiological sign of AD is a decrease in blood flow in the brain [82]. Also, another recent study reported that Polarization-Sensitive Optical Coherence Microscopy could assess amyloidosis based on intrinsic birefringent properties [83]. This finding could open a new venue for detecting Aβ plaques in the retina as another alternative for early diagnosis of AD. It is also essential to determine whether measurements investigated as potential biomarkers are repeatable and reproducible in patients with cognitive impairment and healthy controls to discard that OCT detected changes may be due to disease mechanisms or might be credited to measurement variability or typical progression due to aging and other factors not related to cognitive function decline [22].

The most recent research has identified Aβ deposits in eyes from both transgenic mouse models and human AD [84,85]. In another study, AD transgenic mice were administered curcumin, which binds to amyloid plaques. These studies confirmed the existence of amyloid deposits in the retina preceded amyloid plaque formation in the brain [84,85]. Both studies offer encouragement to the idea of imaging the retina of AD patients, as a potential biomarker that mirrors cerebral amyloid deposition. Cognoptix, Inc., and NeuroVision LLC. have been working on tests to detect amyloid beta plaques in the eye [86,87]. The Sapphire II test (Cognoptix, Inc., Concord, MA, U.S.) is based on detecting amyloid plaques in the crystalline lens using a fluorescent ligand marker applied topically to the inner eyelid of one eye (three ointment applications at home, 2 hours apart) the evening before the procedure. The Retinal Amyloid Index test (NeuroVision, LLC, Sacramento, CA, U.S.) detects amyloid plaques in the retina by administering a curcumin compound orally to the patient and imaging the patient’s retina later with a device that is similar to a conventional retinal imaging scanner. NeuroVision’s and Cognoptix’s tests are promising, but they are not yet long-established as accurate, useful, and concrete using larger data.

### 3.5. OCT Findings in Relation to Advanced Imaging and Electrophysiological Techniques

Studies using the fundus images have revealed that major vessels in the retina are affected in patients with cognitive decline; however, advanced imaging technologies may be able to detect abnormalities in smaller vessels (e.g., capillaries). This potential advantage could make it possible to investigate subtle changes in static vascular parameters (e.g., capillary density) that may correlate with dynamic vascular parameters (e.g., pulse wave velocity and blood pressure) which have also been correlated with declining cognitive function [88,89,90,91]. There may also be the possibility to detect abnormal functional patterns of the retinal neurons by using electroretinography and other visual function tests. Notably, recent studies indicate that electrophysiological-based methodologies could become an alternate approach for the detection of abnormal neuronal activity and networking in the early stage of AD [92]. Interestingly, recent research reported that the scotopic response declined in C57/BL6 mice after subretinal injection of Aβ [93].

The integrity of the retinal tissue is not readily determined by fundus examination in the nonexistence of signs of vascular and structural abnormalities but can be assessed throughout both imaging analyses and functional testing. The confounding similarities between the pathogenesis of age-related macular degeneration (AMD), glaucoma and AD impose a significant challenge to early diagnose AD using changes in the retina. For example, AMD and AD share several pathogenic mechanisms such as oxidative stress and neuroinflammation, and also Aβ has been found to be located in drusen which are common findings in AMD [79]. While some studies have reported a non-significant correlation between retinal structures and AD severity [37,44,46,48,62], most have reported thinning of RNFL and GCL associated with AD severity [20,36,39,40,43,45,47,49,50,51,52,53,59,64,65]. These findings are contradictory, pointing to the need to investigate not only static measures but dynamic metrics that may have a higher probability of being relevant in identifying those at risk. Considering that synaptic loss is one of the earliest functional signs of AD, as well as the best correlate of cognitive function decline [94,95], and that decreased a- and b-wave amplitudes were reported for mice carrying the ApoE-ε4 allele of apolipoprotein [96], ERG exploration could be a valid option to search for diagnostic markers of synaptic dysfunction within the retina that might be indicative of alterations in the brain [97].

Contemplating the above-mentioned findings, a multimodal integrative approach may offer a better venue to identify potential endpoints for neuroretinal impairment in patients with cognitive decline [97,98]. Also, it should be considered that different multimodal tests may be more useful for different stages of cognitive decline. Therefore, longitudinal studies would be important to determine how to best proceed with patient care and follow-up. Provided a clinical correlation between the brain and eye measures is established, screening of eyes in people considered at risk of AD could aid in the elaboration of a complementary low-cost approach for early diagnosis, as well as serving to monitor the effectiveness of developing therapies potentially.

## 4. Conclusions

The identification of retinal biomarkers in AD using OCT remains an area of active research. The main advantages of retinal imaging for diagnosing probable AD include its user-friendliness, low-cost, and the non-invasive nature of examinations. This review has identified a significant gap: the need for a clinically accessible framework to evaluate eye-brain measurements simultaneously across the lifespan. Crucially, this would enable baseline measurements before brain dysfunction happens and might allow the routine collection of retinal function metrics in the future, much like body temperature, pulse rate, and blood pressure measures today. However, successful longitudinal monitoring of eye-brain functional changes entails the establishment of accurate structural-functional baselines of eye-brain vitality before conditions of dysfunction. This framework should facilitate the translation of multimodal eye data into user-friendly metrics of brain function for broader clinical utilization, including the characterization of aging-related confounds. It is also worth noting that the use of big data and open-source models taking advantage of artificial intelligence are a critical step for more integrative, data-driven clinical studies to investigate into all the possible biological features involved, as well as the direct connections among these features. Also, it is crucial to improve the automation, ergonomics and user-friendliness of the designs of OCT technology for the elderly, who very frequently cannot follow instructions and complete an OCT examination successfully, making OCT assessment unreliable in this population.

In the coming decade, the aging research community, especially those investigating preclinical markers through the eye for a pathological cognitive decline, envisages to understanding better what eye-brain measures individually or collaboratively best predict future functional impairment. Also, longitudinal studies with larger sample sizes with a well-design stratified sampling and with a focus on the use of retinal measures by the geriatric population are needed, as this community group continues to grow and medical cost rise. A significant challenge will be to discover specific ocular biomarkers with sufficient sensitivity to reveal preclinical disorder and to monitor progression precisely. Ultimately, researchers will use the information from eye metrics to further examine the effectiveness of interventions in slowing or halting the development of cognitive function disorders.

## Figures and Tables

**Table 1:** Studies evaluating retinal biomarkers in Alzheimer’s disease using optical coherence tomography.

Source	Sample Size	Criteria Used for Selecting Participants		Findings	MRI or CT (Control Group) & ERG (Both Groups)	OCT Platform	Gender (F/M) & Race
		Pathological Group	Control Group				
Santos et al. 2018 [29]	(*n* = 56) older adults with multiple risk factors for AD.	All patients underwent detailed medical screening, patients with AD and MCI according to the NIAAA diagnostic criteria were excluded.		Preclinical stage of AD showed a decrease in macular RNFL volume over a 27-month follow-up interval period, as well as a decrease in ONL and IPL volumes/thickness in the inferior quadrant. Only the RNFL volume was linearly related to neocortical PET amyloid standardized uptake value ratio after controlling for any main effects of age. The magnitude of RNFL volume reduction was correlated with performance on a task of participants’ abilities to efficiently integrate visual and auditory speech information (McGurk effect).	YES (PET with head CT)	Heidelberg Spectralis	26F/48M Race: unknown
Uchida et al. 2018 [30]	(*n* = 24) AD Dementia, (*n* = 22) Amnestic MCI, (*n* = 20) non-AD dementia, (*n* = 22) PD, (*n* = 36) controls	AD diagnosis according to NIAAA criteria.		The outer retinal thickness measures did not show any statistical significance between the groups. However, ellipsoid zone to retinal pigment epithelium volume correlated with cognitive testing scores in all study participants.	YES	Zeiss Cirrus	14F/8M Amnestic MCI 9F/11M non-AD Dementia 10F/12M PD 22F/14M Controls Race: unknown
Lad et al. 2018 [26]	(*n* = 15) MCI, (*n* = 15) mild-moderate AD, (*n* = 18) cognitively normal adults.	AD diagnosis according to the NINCDS-ADRDA criteria. MCI diagnosis according to the NIAAA criteria.		Regional thicknesses of NFL or GCIPL on macular or nerve OCTs did not differ between groups. Multivariate regression analysis identified macular areas with a significant thickening or thinning in NFL and GC-IPL in MCI and AD patients.	NOT ALWAYS	Heidelberg Spectralis	AD: 7F/8M, 14 Caucasian MCI: 8F/7M, 15 Caucasian Controls: 8F/10M, 14 Caucasian
Den Haan et al. 2018 [31]	(*n* = 15) early onset amyloid-positive AD, (*n* = 5) controls.	AD according to NIAAA criteria, evidence of amyloid pathology in CSF and/or amyloid PET.		Total macular thickness correlated with parietal cortical atrophy in both groups.	YES (MRI)	Heidelberg Spectralis	7F/8M AD 7F/8M Controls Race: unknown
Sánchez et al. 2018 [32]	(*n* = 24) probable AD (*n* = 92) probable amnestic MCI (*n* = 414) controls	Diagnosis according to the MMSE, 7-minute tests and the Hospital Anxiety and Depression Scale		The multivariate adjusted analysis revealed no significant differences in mean overall, or local RNFL thickness between AD, MCI and control groups. These results do not support the usefulness of peripapillary RNFL analysis as a marker of MCI/AD.	NOT ALWAYS	Topcon Maestro	74.0% women AD 56.2% women MCI 67.7% women Controls Race: unknown
Poroy et al. 2018 [33]	(*n* = 21) AD (*n* = 25) controls	AD diagnosis according to DSM-V		Foveal thickness and volume were significantly higher in AD patients than in controls. Compared to controls, peripapillary RNFL and other macular region measurements of AD patient were not statistically different.	N/A	Zeiss Stratus	15F/6M AD 18F/7M Controls Race: unknown
Cunha et al. 2017 [19]	(*n* = 50) patients with mild AD, (*n* = 152) patients without AD (age-matched patients), (*n* = 50) elderly patients without AD (over 78 years of age)	Each patient sent by the neurology department underwent visual acuity, IOP, mean arterial pressure assessment.		AD patients showed a significant choroidal thinning even when compared with elderly subjects. The reduction of CT may aid in the diagnoses of AD, probably reflecting the importance of vascular factors in their pathogenesis.	NO	Heidelberg Spectralis	34F/16M AD 97F/55M Controls 33F/17M Controls Race: unknown
Ferrari et al. 2017 [34]	(*n* = 39) AD, (*n* = 17) FTD, (*n* = 27) MCI, (*n* = 49) controls	Albert criteria for MCI, NINCDS-ADRDA criteria for AD, Rascovsky criteria for FTD.		GCL-IPL was significantly correlated with raw MMSE in AD.	N/A	Heidelberg Spectralis	24F/13M AD 9F/8M FTD 15F/12M MCI 26F/23M Controls Race: unknown
Kwon et al. 2017 [35]	(*n* = 15) AD (*n* = 15) MCI (*n* = 15) controls	AD diagnosis according to the MMSE, CDR and Activities of Daily Living Scales. MCI according to the Petersen criteria.		Mean RNFL thickness was lower in the AD group than in the MCI group. RNFL thickness in the superior quadrant was lower in AD patients compared to controls.	YES (MRI)	Zeiss Cirrus	Gender and Race unknown
Trebbastioni et al. 2017 [27]	(*n* = 39) AD (*n* = 39) age matched controls subjects	AD diagnosis according NINCDS -ADRDA criteria and MMSE/CDR.	Controls underwent physical and neurological assessment, standard laboratory tests, serum vitamin B12, folate and thyroid hormone assays.	Choroidal thickness in patients with AD showed a rate of thinning greater than what could be expected during the natural course of aging.	NO	Heidelberg Spectralis	21F/18M AD 22F/17M Controls Race: unknown
Mutlu et al. 2017 [36]	Included 2124 persons from the Rotterdam Study who had gradable retinal OCT images and brain MRI scans.	Cognitive test battery including the MMSE and the Geriatric Mental State Schedule organic level Dementia-screening was done using the MMSE and the Geriatric Mental State Schedule (GMS) organic level.		Thinner RNFL, GCL and IPL were associated with smaller grey matter and white matter volume. Thinner RNFL and GCL were associated with worse white matter microstructure. No association between retinal sublayer thickness and white matter lesion volumes, cerebral microbleeds or lacunar infarcts.	YES (MRI)	Topcon 3D OCT 1000/2000	1190F/934M Race: unknown
Snyder et al. 2016 [37]	Cognitively normal adults, all of whom have a parent with AD and subjective memory complaints (SMC, *n* = 63)	All participants underwent a detailed medical screening interview. Exclusion criteria were a diagnosis of MCI or AD, history of neurological or psychiatric disorder, any significant systemic illness or unstable medical condition used MMSE and DASS.		Trend toward a selective volume increase in the IPL (which is rich in cholinergic activity) of the retina in Aβ+ relative to Aβ− participants, and IPL volume was correlated with the surface area of retinal inclusion bodies.	NO	Heidelberg Spectralis	39F/24M Race: unknown
Gimenéz Castejon et al. 2016 [38]	The total number of valid patients was (*n* = 57), (*n* = 24) of them diagnosed with SMC and (*n* = 33) with MCI and (*n* = 25) control group.	The diagnosis of MCI was based on the standards of the DSM-IV. For the SMC patients, the same cognitive screening as that for the MCI patients was performed.		Statistically significant differences have been found in the macular thickness of the control group and for both MCI and SMC patients.	NO	Zeiss Cirrus	12F/12M SMC, 10F/15M MCI 15F/18M Controls Race: unknown
Choi et al. 2016 [39]	(*n* = 42) patients with AD, (*n* = 26) with MCI, (*n* = 66) normal elderly controls.	The subjects with AD met the criteria for dementia according to the DSM-IV, as well as the criteria for probable AD established by the NIAAA. The diagnosis of MCI was in accordance with Petersen et al.’s criteria, listed as follows: (1) subjective memory complaints corroborated by an informant; (2) objective memory decline, as defined by a delayed recall score on the Seoul Verbal Learning test (SVLT) less than 1.5 standard deviations (SD) below the age- and education-adjusted normative means; (3) normal general cognitive function, as defined by CDR scale of 0.5, and MMSE scores more than 1.5 SD below the age- and education-adjusted normative means; (4) normal functional activities; and (5) lack of a dementia diagnosis.		The Clinical Dementia Rating Scale-Sum of Boxes (CDR-SB) score presented negative relationships with the average GCIPL thickness and the GCIPL thickness in the superotemporal, superonasal, and inferonasal sectors. The composite memory score exhibited significant positive associations with the average GCIPL thickness and the GCIPL thickness in the superotemporal, inferonasal, and inferotemporal sectors. The temporal RNFL thickness, the average and minimum GCIPL thicknesses, and the GCIPL thickness in the inferonasal, inferior, and inferotemporal sectors at baseline were significantly reduced in MCI patients who were converted to AD compared to stable MCI patients. The change of CDR-SB from baseline to 2 years exhibited significant negative associations with the average and minimum GCIPL thicknesses as well as GCIPL thickness in the superotemporal, superior, superonasal, and inferonasal sectors at baseline. Data suggest that macular GCIPL thickness represents a promising biomarker for monitoring the progression of MCI and AD.	YES	Zeiss Cirrus	38F/4M AD 16F/10M MCI 38F/28M Controls Race: unknown
Knoll et al. 2016 [40]	Participants with a clinical diagnosis of aMCI and Cognitively normal control participants (*n* = 17/17)	The diagnosis of MCI based on research diagnostic criteria including the following: scores falling 2 or more standard deviations below the mean on neuropsychological tests MMSE within a battery used across the National Institute on Aging ADC programs (i.e., the Uniform Data Set (UDS)) and the absence of impairments of activities of daily living as corroborated by a study partner.	Control subjects had no abnormal test scores on the UDS battery and all had normal activities of daily living as reported by their study partners.	No statistically significant difference in optical coherence tomography (OCT) between aMCI subjects and controls, but uncovered that OCT thickness was significantly (inversely) related to cognitive scores. The meta-analysis showed statistically significant thinning in MCI subjects compared with controls.	NO	Heidelberg Spectralis	13F/4M aMCI 13F/4M Controls Race: African American 5 aMCI/5 Controls; Caucasian 12 aMCI/12 Controls
Liu et al. 2016 [41]	Participants group difference in WM microstructure in (*n* = 65) no cognitive impairment (NCI), (*n* = 68) CIND, and (*n* = 47) AD and the WM-GC-IPL association in a subset of 124 subjects who passed the retina imaging quality control (*n* = 180)	All subjects underwent MMSE, MoCA, CDR, Geriatric Depression Scale, the informant questionnaire on cognitive decline, and a formal neuropsychological battery. Subjects were also assessed for neuroimaging evidence of significant cerebrovascular disease.		Within those participants with OCT scans of sufficient quality for analysis, GC-IPL was significantly thinner in CIND than in NCI.	YES	Zeiss Cirrus	NCI: 29F/36M CIND: 33F/35M AD: 30F/17M Chinese:Malay:Indian:Mix:Others NCI 60:2:3:0:0 CIND 55:2:11:0:0 AD 38:6:1:1:
Cunha et al. 2016 [20]	A total of 45 eyes from (*n* = 24) patients with AD and 48 eyes from (*n* = 24) healthy controls.	Each patient underwent a full neurological examination MMSE and MRI of the brain to rule out alternative diagnoses.		Most OCT peripapillary RNFL and macular full-thickness and segmented inner retinal layers parameters were reduced in AD compared to controls. Average, superior and inferior quadrant RNFL thickness parameters and all but one of the nine full-thickness macular measurements were significantly reduced in AD compared to controls. The segmented layers, GCL+ and GCL++ were significantly reduced in AD eyes. Significant correlation between most OCT parameters and MMSE scores, particularly in macular thickness.	YES	Topcon 3D OCT-2000	16F/8M AD group 15F/9M Controls Race: unknown
La Morgia et al. 2016 [42]	(*n* = 21) AD, (*n* = 74) age-matched control subjects.	Included patients with a diagnosis of AD according to NINCDS-ADRDA criteria at mild–moderate stage (MMSE>11). Absence of cognitive dysfunction was ascertained in controls.		Age-related optic neuropathy in AD by OCT, with a significant reduction of RNFL thickness, more evident in the superior quadrant.	NO	Zeiss Stratus	10F/11M AD 43F/31M Controls Race: unknown.
Garcia Martin et al. 2016 [43]	Patients with AD (*n* = 150) and age-matched healthy controls (*n* = 75)	Inclusion criteria were AD diagnosis according to the NINCDS-ADRDA, MMSE and DSM-IV criteria.	The controls were family members or caregivers of the patients or health workers. All subjects underwent neurologic and neuro-ophthalmologic evaluations.	The segmentation application revealed ganglion cell and retinal layer atrophy in patients with AD compared with controls, especially in the inner layers of patients with long disease duration. Ganglion cell layer reduction was associated with increased axonal damage and may predict greater disease severity.	NO	Heidelberg Spectralis	84F/66M AD group 42F/33M Controls Race: unknown
Pillai et al. 2016 [44]	AD dementia (*n* = 21), amnestic MCI (*n* = 21), non-AD dementia (*n* = 20), PD (*n* = 20), (*n* = 34) age-/sex-matched controls.	As part of neurocognitive testing, study participants completed the MoCA, Logical Memory subtest of the Wechsler Memory Scale—Fourth Edition, Hopkins Verbal Learning Test–Revised phonemic. And semantic verbal fluency, and Trail Making Test (parts A and B).		Among SD-OCT measures, the RNFL, GCL, and MV were not significantly different across all groups. Using all SD-OCT measures in a mixed-effect model did not identify any significant. Differences between the groups. The RNFL thickness measures analysis by group and quadrant also did not show any statistically significant difference between the groups.	YES	Zeiss Cirrus	13F/8M AD 12F/9M Amnestic MCI 11F/9M NonAD Dementia 11F/9M PD 20F/14M Controls Race: unknown
Liu et al. 2015 [45]	A total of 93 cognitive impaired subjects comprising 26 MCI, 24 mild AD patients, 24 moderate AD patients, 19 severe AD patients, and 39 age-matched controls (*n* = 39)	All AD patients were diagnosed according to the NINCDS-ADRDA and DSM-IV criteria.	The criteria for controls were: (1) no memory complaints; (2) MMSE scores above 28.	RNFL degeneration is paralleled with dementia progression. Owing to its non-invasive and cost-effective nature, monitoring RNFL thickness may have a value in assessing disease progression and the efficacy of any treatments. The thickness of RNFL in the superior quadrant and total mean values are gradually and significantly decreased from MCI to severe AD when compared to that in the controls. There is also a significant reduction of the RNFL in the inferior quadrant in severe AD patients.	NO	Zeiss Stratus	52F/41M AD group 22F/17M Controls Race: unknown
Gao et al. 2015 [46]	A total of 72 subjects, comprising 25 AD patients, 26 MCI patients (*n* = 51), and 21 healthy individuals (controls) (*n* = 21)	All subjects underwent complete neurological MMSE scale and physical examination, ophthalmic examination, laboratory examination of body fluids, neuroimaging evaluation, and psychometric testing to rule out alternative diagnoses. Neuroimaging examination either through MRI or CT was adopted to exclude participants suffering from other neurological or non-neurological diseases that may influence the study results.		Retinal Degeneration in AD and MCI patients results in decreased thickness of the RNFL, and reduced macular volume in AD and MCI patients. However, there seems to be no correlation between these changes and the severity of dementia.	YES	Zeiss Cirrus	24F/26M AD/MCI 7F/14M Controls Race: unknown
Cheung et al. 2015 [47]	Cognitively normal controls (*n* = 123), patients with AD (*n* = 100), MCI (*n* = 41)	All patients underwent clinical and neuropsychiatric assessment. CT or MRI was reviewed as part of the diagnostic process. AD patients fulfilled the DSM-IV criteria for dementia syndrome (Alzheimer’s type) and NINCDS-ADRDA criteria for probable or possible AD.		AD patients compared with cognitively normal controls had significantly reduced GC-IPL thicknesses in all six (superior, superonasal, inferonasal, inferior, inferotemporal, and superotemporal) sectors and reduced RNFL thickness in superior quadrant. Patients with MCI also had significantly reduced GC-IPL thicknesses compared with controls. Supports the link between retinal ganglion cell neuronal and optic nerve axonal loss with AD, and suggest that assessment of macular GC-IPL can be a test to detect neuronal injury in early AD and MCI.	YES	Zeiss Cirrus	85F/56M AD/MCI 56F/67M Controls Race: Chinese/Malay/Indian: 108/18/15 in AD/MCI group, and 123/0/0 control group
Salobrar-Garcia et al. 2015 [48]	(*n* = 23) patients with mild AD (*n* = 28) age-matched control subjects.	These patients, according to the NINCDS-ADRDA, MMSE and DSM-IV criteria had mild cognitive impairment according to the Clinical Dementia Rating scale.		Eyes of patients with mild-AD patients showed no statistical difference in peripapillary RNFL thickness; however, sectors 2,3,4,8,9, and 11 of the papillae showed thinning, while in sectors 1,5,6,7, and 10 there was thickening. Total macular volume and RNFL thickness of the fovea in all four inner quadrants and in the outer temporal quadrants proved to be significantly decreased.	NO	Topcon 3D OCT-1000	14F/9M AD 19F/9M Controls Race: Caucasian
Octem et al. 2015 [49]	(*n* = 35) patients with AD, (*n* = 35) patients with MCI, (*n* = 35) healthy volunteers	Cognitive assessment was done with the standardized MMSE and MoCA test		No significant differences of RNFL were found between the MCI and the AD groups. Significant correlation was found between MMSE scores and the RNFL values. Significant thinning in RNFL along with age was detected.	NO	Zeiss Cirrus	23F/12M AD 20F/15M MCI 23F/12M Controls Race: unknown
Bayhan et al. 2015 [28]	(*n* = 31) AD (*n* = 30) age =matched controls	The diagnosis of probable AD was determined by referring neurologists according to the NINCDS-ADRDA and MMSE.	The control subjects also underwent a detailed neurological examination to rule out the presence of cognitive impairment.	Reduced choroidal and macular ganglion cell complex thicknesses in AD	YES	Zeiss Stratus	14F/17M AD 14F/16M Controls Race: unknown
Eraslan et al. 2015 [50]	(*n* = 18) normal tension glaucoma (NTG), (*n* = 20) AD, (*n* = 20) control subjects	Diagnosis was based on NINCDS/ADRDA.	Each patient underwent neurological examination.	There was a significant reduction in peripapillary RNFL thickness and macular GCC thickness and a significant increase in the global loss volume (GLV) rate in both the NTG and AD patients when compared to the control subjects. The statistical evaluation showed no difference in any RNFL or GCC parameters between the AD and NTG groups. There was a negative correlation between disease duration and average RNFL and GCC thicknesses and a positive correlation between duration and GLV in the AD group.	NO	RTVue 100	10F/8M NTG group 13F/7M AD group 14F/6M Controls Race: unknown
Ascaso et al. 2014 [51]	(*n* = 18) patients with AD, (*n* = 21) aMCI, (*n* = 41) healthy controls	Diagnosis was based on DSM IV, MMSE scale, of MCI, used Winblad criteria.	Each patient underwent full neurologic examination to rule out the presence of dementia or cognitive impairment.	RNFL was thinner in MCI patients compared with controls, and it was also thinner in AD patients compared with MCI patients and controls. With regard to the macular measurements in mm^3^, MCI patients had the greatest macular volume in comparison with AD patients and controls. In turn the controls had greater macular volume than AD patients. The decreased RNFL thickness in MCI and AD patients suggests loss of retinal neurons and their axons.	NO	Zeiss Stratus	21F/18M AD/MCI 21F/20M Controls Race: Hispanic
Garcia-Martin et al. 2014 [52]	Twenty patients with mild AD (*n* = 20), (*n* = 28) matched control subjects	The AD patients met the criteria for AD according to the NINCDS-ADRDA, MMSE and DSM-IV criteria, having MCI according to the CDR scale.	All the subjects had a complete ophthalmologic examination, including VA, refraction, anterior and posterior segment biomicroscopy, IOP measurement, dilated fundus examination, and OCT.	Mild AD patients, compared with a control group, had a statistically significant decrease in RNFL thickness, of some macular regions and in the total macular volume.	NO	Topcon 3D OCT-1000	12F/8M AD group 19F/9M Controls Race: Caucasian
Polo et al. 2014 [53]	(*n* = 70) with AD, (*n* = 70) sex- and age-matched healthy subjects	Inclusion criteria were confirmed AD diagnosis; Diagnosis of AD was determined by neurologists according to the NINCDS-ADRDA, DSM-IV and MMSE criteria.	Healthy controls had no evidence of disease of any nature, including neurologic disorders by interview.	SD-OCT is a valid and reliable technique for detecting subclinical RNFL and retinal atrophy in AD, especially using the Nsite Axonal application. RNFL thickness decreased with disease duration.	NO	Zeiss Cirrus/Heidelberg Spectralis	40F/30M AD group 40F/30 M Controls Race: Hispanic
Gharbiya et al. 2014 [18]	42 eyes of (*n* = 21) patients (mean age, 73.1 ± 6.9 years) with a diagnosis of mild to moderate AD, 42 eyes of (*n* = 21) age-matched control subjects (mean age, 70.3 ± 7.3 years)	All the subjects underwent neuropsychological (MMSE, ADAS-Cog, and CDR) and ophthalmological evaluation. The SD-OCT images of the choroid were obtained by EDI modality. Choroidal thickness was assessed by manual measurement. The following parameters, measured automatically by the OCT software, were also analyzed for each eye: 1-mm central subfield retinal thickness, peripapillary RNFL thickness.		Compared with healthy subjects, patients with AD showed a significant reduction in choroidal thickness.	NO	Zeiss Stratus	Gender and Race unknown
Shen et al. 2014 [54]	(*n* = 75) older adults were included in the study, (*n* = 52) participants had normal cognition (NC), (*n* = 23) participants were diagnosed with MCI.	Cognitive function was evaluated by the Repeatable Battery for the Assessment of Neuropsychological Status on the same day of the optical examination.		Found that nasal quadrant RNFL thickness was positively associated with episodic memory scores in the participants with normal cognition	NO	Zeiss Stratus	34F/41M Race: unknown
Shi et al. 2014 [55]	Participants categorized as stable participants whose cognitive status remained unchanged (*n* = 60) and converted participants whose cognitive status deteriorated, which was diagnosed by DSM-VI (for AD) and Petersen’s definition (for MCI) (*n* = 18)	The participants were first screened for dementia by using the Chinese version of the MMSE, Chinese version of Activities of the Daily Living Scale, and the Chinese version of the RBANS		The reduction in the inferior quadrant of RNFL thickness might indicate a higher risk for the old adults to develop cognitive deterioration.	NO	Zeiss Cirrus	34F/26M Stable group 10F/8M Converted group Race: unknown
Kromer et al. 2014 [56]	(*n* = 22) AD, (*n* = 22) age-gender-matched healthy subjects	Patients with mild to moderate AD and a cognitively healthy age-matched control subjects were recruited from the Memory Clinic of the Department of Geriatric Psychiatry of the Central Institute of Mental Health, Mannheim, Germany MMSE scale		Patients with AD showed a significant decrease in RNFL thickness in the nasal superior sector compared to the control group (101.0 ± 18.18 μm versus 122.8 ± 28.08 μm; *p* < 0.0001). In all other sectors, independently of disease duration, no significant difference in RNFL thickness compared to controls was detected.	YES	Heidelberg Spectralis	14F/8M AD 15F/7M Controls Race: unknown
Larrosa et al. 2014 [57]	(*n* = 151) AD, (*n* = 61) age-matched healthy subjects	The AD diagnosis was determined by neurologists according to the NINCDS-ADRDS, DSM IV and MMSE criteria.		Reduced peripapillary RNFL thickness using linear discrimination function in AD	NO	Zeiss Cirrus and Heidelberg Spectralis	95F/56M AD 38F/23M Controls Race: unknown
Bambo et al. 2014 [58]	(*n* = 56) AD (*n* = 56) healthy controls	Confirmed AD diagnosis; and MMSE scale		Reduced peripapillary RNFL thickness in superior and inferior quadrants in AD.	NO	Zeiss Cirrus	Gender and Race unknown
Moreno-Ramos et al 2013 [59]	AD (*n* = 10), Dementia with Lewy bodies (*n* = 10), Dementia associated with Parkinson’s disease (*n* = 10), Cognitively normal age-matched controls (*n* = 10)	Patients with AD met the NINDS-ADRDA criteria of probable AD, MMSE scale while patients with dementia with Lewy bodies fulfilled McKeith’s criteria. With respect to the diagnosis of the patients with dementia associated with Parkinson’s disease, followed the recommendations of the Movement Disorders Society Task Force.	All controls underwent a detailed neurologic and neuropsycholo-gical examination	The thickness of the RNLF correlated significantly (*p* < 0.001) with both the MMSE and the Mattis Dementia Rating Scale scores in all types of dementia; that is to say, the greater the cognitive deterioration, the greater the reduction of thickness of the RNLF. The findings from this study show that retinal involvement measured by optical coherence tomography may also be present in non-AD dementias.	NO	Topcon 3D OCT-1000	4F/6M Controls 4F/6M Alzheimer’s 5F/5M Dementia with Lewy bodies 4F/6M Dementia associated with Parkinson’s disease Race: unknown
Marziani et al. 2013 [60]	(*n* = 21) AD patients (*n* = 21) healthy subjects	Inclusion criteria were AD diagnosis according to the NINCDS-ADRDA	Each patient underwent full neurologic examination	RNFL and GCL in AD patients was reduction	NO	RTV-ue100 and Heidelberg Spectralis	17F/4M AD group 16F/5M Controls Race: unknown
Kirbas et al. 2013 [61]	(*n* = 40) patients with early untreated AD (mean age, 69.3 ± 4.9 years) (*n* = 40) healthy controls (mean age, 68.9 ± 5.1 years)	Each patient underwent full neurologic examination, MMSE scale and brain MRI to exclude alternative diagnoses.		Thickness of RNFL in patient with AD was lower than that of controls. This suggests that SD- OCT has the potential to be used in the early diagnosis of AD as well as in the study of therapeutic agents.	YES	Heidelberg Spectralis	18F/22M AD group 20F/20M Controls Race: unknown
Moschos et al. 2012 [17]	(*n* = 30) patients with AD (*n* = 30) age and sex matched healthy controls	Diagnosis was based on NINCDS-ADRDA		Patients with AD, even without visual failure there was a decrease in macular and RNFL thickness, as well as a decrease of the electrical activity of the macula	NO ERG/VEP	Zeiss Stratus	15F/15M AD 15F/15M Controls
Kesler et al. 2011 [62]	AD diagnosis according to DSM-IV criteria-(*n* = 54) subjects. Cognitively healthy age-matched volunteers were also examined as control subjects (*n* = 24)	Ophthalmological evaluation included VA, IOP, slit lamp biomicroscopy and visual field examination. OCT measurements were performed by another ophthalmologist. All researchers were familiar with study protocol, but both ophthalmologists were blinded to cognitive status and diagnosis.		The total RNFL thicknesses were significantly different between the three groups: the RNFL was significantly thinner in the MCI Group compared to controls, as well as when the AD group was compared to MCI.	NO	Zeiss Stratus	Gender and Race unknown
Lu et al. 2010 [63]	(*n* = 22) AD group, (*n* = 22) healthy controls	AD patients with clinical mild or moderate dementia were diagnosed by the AD group neurologists in the department of Neurology, Xuanwu Hospital, according to the NINCDS-ADRDA		The RNFL thickness of AD patients were much thinner especially in supra-retina and infra-retina, while no difference was found in the other retinal area.	NO	Zeiss Stratus	12F/10M AD group 12F/10M Controls
Paquet et al. 2007 [64]	(*n* = 15) healthy, aged-matched subjects, (*n* = 23) MCI patients, (*n* = 14) mild AD patients, (*n* = 12) moderate to severe AD patients enrolled.	AD patients fulfilled the NINCDS-ADRDA criteria, and MMSE scale. Control subjects and MCI patients had no neurological or ophthalmologic diseases.		The results show that RNFL thickness is statistically reduced in patients with MCI, mild AD or moderate to severe AD compared to controls. In addition, no statistical difference was found between the results in MCI patients and mild AD patients. The RNFL seems to be involved early during the course of amnestic MCI and OCT tests could be carried out in patients with cognitive troubles.	NO	Zeiss Stratus	13F/2M Controls 15F/8M MCI 9F/5M Mild AD 6F/6M Severe AD Race: unknown
Iseri et al 2006 [65]	28 eyes of (*n* = 14) patients with AD, 30 eyes of (*n* = 15) age-matched control subjects.	AD set by the NINCDS-ADRDA, DSM-IV and MMSE scale. AD patients had mild and moderate cognitive impairment according to the CDR scale.		The peripapillary and macular RNFL thickness in all quadrants and positions of AD patients were thinner than in control subjects. The mean total macular volume of AD patients was significantly reduced as compared with control subjects. Total macular volume and MMSE scores were significantly correlated. No significant difference was found in the latency of the VEP P100 of AD patients and control subjects.	NO VEP	Zeiss Stratus	8F/7M AD 8F/7M Controls Race: unknown
Parisi et al. 2001 [66]	(*n* = 17) subjects with AD, (*n* = 14) age-matched controls subjects.	The AD patients met the criteria for AD according to the NINCDS-ADRDA, DSM-IV and MMSE, MRI was performed.		AD patients have reduction of NFL thickness. This morphological abnormality is related to a retinal dysfunction as revealed by abnormal PERG responses.	YES PERG	Zeiss Stratus	Gender and Race unknown

Abbreviations: AD—Alzheimer’s disease, Aβ—amyloid beta, CDR—clinical dementia rating, CIND—cognitive impairment without dementia, CT—computer tomography, DSM—diagnostic and statistical manual of mental disorders, FTD—frontotemporal dementia, GC-IPL—ganglion cell/inner plexiform layer, GCL—ganglion cell layer, IOP—intraocular pressure, MCI—mild cognitive impairment, MoCA—montreal cognitive assessment, MMSE—mini-mental state examination, MRI—magnetic resonance imaging, NFL—nerve fiber layer, NIAAA—National Institute on Aging/Alzheimer’s Association, NINCDS-ADRDA—National Institute of Neurological and Communicative Disorders and Stroke-Alzheimer’s Disease and Related Disorders Association, OCT—optical coherence tomography, ONL—outer nuclear layer, PD—Parkinson’s disease, PET—positron emission tomography, RNFL—retinal nerve fiber layer, OCT—optical coherence tomography, SD-OCT—spectral-domain OCT, VA—visual acuity, ERG—electroretinogram, VEP—visual evoked potential, VER—visual evoked response, PERG—pattern electroretinogram, F—female, M—male, N/A—non-available, RBANS—repeatable battery for the assessment of neuropsychological status, ADAS-Cog—Alzheimer’s disease assessment scale-cognitive subscale, GLV—global loss volume, UDS—uniform data set, aMCI—agnostic mild cognitive impairment, SD—standard deviation, SVLT—Seoul verbal learning test, CDR-SB—clinical dementia rating scale-sum of boxes, GMS—geriatric mental state schedule, SMC—subjective memory complaints, NCI—no cognitive impairment, NTC—normal tension glaucoma, NC—normal cognition, WM—white matter, DASS—depression anxiety stress scale, EDI—enhanced depth imaging.
